# Long-term Safety and Efficacy of the Derivo Embolization Device in a Single-center Series

**DOI:** 10.1007/s00062-024-01423-1

**Published:** 2024-05-30

**Authors:** Lukas Goertz, David Zopfs, Jonathan Kottlors, Jan Borggrefe, Lenhard Pennig, Marc Schlamann, Christoph Kabbasch

**Affiliations:** 1 Faculty of Medicine and University Hospital, Department of Radiology and Neuroradiology, University of Cologne, Kerpener Straße 62, 50937 Cologne, Germany; 2 Department of Radiology, University Hospital Minden, Minden, Germany

**Keywords:** Acandis, Aneurysm, Endovascular, Low-profile stent, Outcome

## Abstract

**Purpose:**

This study analyzes the long-term clinical and angiographic outcomes of the Derivo Embolization Device (DED), an advanced flow diverter device with an electropolished surface, for the treatment of intracranial aneurysms.

**Methods:**

A consecutive series of 101 patients (mean age: 58 years, 72% female) treated with the DED for 122 aneurysms at a single center between 2017 and 2023 was retrospectively analyzed for major (change in National Institutes of Health Stroke Scale [NIHSS] score ≥ 4 points) and minor (change in NIHSS score < 4 points) neurological events, procedural morbidity (increase of at least one point on the modified Rankin Scale), and angiographic results.

**Results:**

There were 14 (11%) recurrent aneurysms, 15 (12%) ruptured aneurysms, 26 (21%) posterior circulation aneurysms and 16 (13%) fusiform or dissecting aneurysms. Device deployment failed in 1 case (1%). Procedure-related symptomatic procedural complications consisted of 2 (2%) major events (1 major stroke and 1 vessel perforation with intracranial hemorrhage and infarction) and 6 minor events (6 minor strokes). Procedural morbidity was 5%. There were no late ischemic or hemorrhagic events during follow-up. Complete and favorable aneurysm occlusion was achieved in 54% (40/74) and 62% (46/74) at a mean of 5 months, 71% (27/38) and 87% (33/38) at a mean of 12 months, and 76% (25/33) and 97% (32/33) at a mean of 35 months, respectively.

**Conclusion:**

The results demonstrate progressive aneurysm occlusion beyond 12 months after DED implantation with an almost 100% favorable occlusion rate. Procedural morbidity was low and there were no late complications.

## Introduction

Since the introduction of the Pipeline Embolization Device (PED, Covidien, Mansfield, MA, USA) in 2008, there has been a growing preference for flow diverters (FDs) in the treatment of intracranial aneurysms, especially those that are wide-necked, large, and fusiform, which present challenges to conventional endovascular treatment [1–4]. Numerous studies have demonstrated high occlusion rates after FD treatment with an acceptable safety profile [5–7]. However, potential disadvantages of flow diverter therapy include the risk of delayed rupture, ischemic stroke, and the latency period required for complete aneurysm occlusion [8–10].

Over the past decade, FDs have evolved with continuous refinement, introducing novel devices for improved deployment and procedural safety. While the safety and efficacy of the PED has been extensively documented, there is limited experience with newer devices [8, 11–13].

The Derivo Embolization Device (DED, Acandis, Pforzheim, Germany) is an advanced FD with antithrombotic surface modification designed to improve procedural safety. Previous studies have shown a promising short- and medium-term safety and efficacy profile, but long-term follow-up data remain limited [14–16].

This single-center series provides comprehensive insights into the mid- and long-term clinical and angiographic outcomes in consecutive patients treated with DED, reflecting real-world scenarios.

## Methods

This is a retrospective, observational, single-arm study that included consecutive patients treated with DEDs for intracranial aneurysms from October 2017 to September 2023. No exclusion criteria were applied, and the local ethics committee approved the anonymous collection of patient data. Informed consent was not required for this retrospective observational study.

### Procedure

The DED is an advanced, surface-modified FD with a thin layer of titanium oxides and titanium oxynitrides (BlueXide®) which is applied by a combined electropolishing and annealing process. This surface modification is designed to minimize friction during deployment and improve the thrombogenic profile. The DED 2 is also available with an additional antithrombotic coating (Heal®), however, these devices were not included in the present study. The DED 1 had three additional radiopaque markers at each end, while the DED 2 omitted this feature due to a higher percentage of platinum in the nitinol composite microwires, making the entire device contour visible under fluoroscopy. The DED is available in diameters from 2.5 to 8 mm, allowing treatment of vessels from 1.5 to 8.0 mm in diameter. In this study, both the DED 1 and DED 2 of all available diameters were used.

All patients were treated under general anesthesia via the transfemoral approach. Elective cases received an initial intravenous heparin bolus (5000 IU), followed by additional aliquots of 1000 IU/h until completion of the procedure. A triaxial system introduced an 8F guiding catheter into the internal carotid artery or a 6F guiding catheter into the vertebral artery. DEDs were delivered through 0.017’’ –0.039’’ microcatheters, depending on the device diameter. Selective balloon angioplasty was used to improve wall apposition. Coils and other materials were used at the discretion of the neurointerventionalist. Successful device deployment included complete DED coverage of the aneurysm neck, ensuring complete wall apposition.

### Antiplatelet Regimen

For planned procedures, patients were prescribed 100 mg acetylsalicylic acid (ASA) and 75 mg clopidogrel for 5–7 days prior to the procedure. Assessment of platelet inhibition included ASA and P2Y12 (Verify Now, Accumetrics, San Diego, CA, USA) assays. Adequate platelet inhibition was defined as levels between 350–550 ARU (ASA Response Units) for ASA and 30–60% for clopidogrel. In cases of inadequate response, dose escalation (e.g., clopidogrel 150 mg/d) or substitution with prasugrel (60 mg bolus, 10 mg/d) was performed. In emergency situations, tirofiban (Aggrastat, Merck, West Point, PY, USA) was administered before DED placement and continued for 16–24 h after the procedure, followed by clopidogrel (300 mg) and aspirin (250 mg) loading. Subsequent post-interventional antiplatelet therapy consisted of continuous aspirin (100 mg/d) and either clopidogrel (75 mg/d) or prasugrel (10 mg/d) for 4 months.

### Data Collection

Patient characteristics and procedural details were obtained by chart review, including details of previous aneurysm interventions, rupture status, number of DEDs used, DED type (DED 1 vs. DED 2), DED diameter, use of adjunctive endovascular techniques, complications, and functional outcomes. All clinically relevant technical events, whether symptomatic or asymptomatic, were documented. The severity of neurological events was assessed using the National Institutes of Health Stroke Scale (NIHSS), with a major event defined as a change in NIHSS score of ≥ 4 points. Functional outcome was measured by the modified Rankin Scale (mRS), and morbidity was defined as an increase of at least one point at discharge compared to baseline. Post-interventional magnetic resonance imaging (MRI) with time-of-flight (TOF) and contrast-enhanced angiography was performed if the patient presented with new neurological symptoms or in complex cases at the discretion of the interventionalist. Preoperative 4‑vessel digital subtraction angiography (DSA) was used to determine aneurysm characteristics, including location, size, neck width, dome-to-neck (D/N) ratio, and parent artery diameter at the proximal and distal landing zone. The ratio of the DED diameter and the mean of proximal and distal parent artery diameter was also calculated.

Angiographic follow-up (FU) included DSA at 6 and 24 months post-treatment, with individual FU periods thereafter based on occlusion status. A minority underwent magnetic resonance angiography (MRA) beyond the 6‑month FU. Short-term FU was 1–6 months post-treatment, medium-term was 7–18 months, and long-term was > 18 months. Aneurysm occlusion was graded according to the O’Kelly-Marotta (OKM) grading scale: A = total filling (> 95%), B = subtotal filling (5–95%), C = entry remnant (< 5%), and D = complete occlusion [17]. OKM C and D were subsumed as favorable occlusion. Immediate contrast retention after FD implantation was graded as 1 = no stasis, 2 = moderate stasis, and 3 = severe stasis. In-stent stenosis was graded as mild (< 25%), moderate (25–50%) or severe (> 50%) [18].

### Statistical Analysis

Categorical variables are presented as numbers and percentages, and group comparisons were made using the chi-square and Fisher’s exact tests, as appropriate. Continuous variables are presented as mean and standard deviation, and group comparisons were performed using the unpaired two-tailed t‑test and the Mann-Whitney U‑test, depending on normality, which was tested with the Shapiro-Wilk test. In the univariate analysis, patient age, sex, aneurysm location, aneurysm type, aneurysm morphology, aneurysm size, parent artery diameter, DED type (DED 1 vs. DED 2), DED diameter, DED-to-parent artery ratio, and use of additional devices were considered as potential predictors of immediate aneurysm sac occlusion, occurrence of minor or major stroke, and favorable occlusion at follow-up. Factors predictive of each outcome measure in the univariate analysis (*p* < 0.1) were entered into a binary logistic stepwise regression model to determine if they were independently associated with the outcome measures. Statistical analysis was performed using SPSS software (IBM SPSS Statistics for Windows, Version 25.0, Armonk, NY, USA), and significance was set at a *p*-value < 0.05.

## Results

### Patient and Aneurysm Characteristics

The study cohort consisted of 101 patients treated for 122 aneurysms in 103 procedures. The mean age of the patients was 57.8 ± 13.7 years (range: 26–90 years), 73 (72.3%) were female and 28 (27.7%) were male.

The most common aneurysm locations were the paraophthalmic internal carotid artery (ICA) in 67 (54.9%) cases, the posterior communicating artery (Pcom) in 19 (15.6%), the basilar artery (BA) in 11 (9.0%), and the vertebral artery (VA) in 10 (8.2%). DEDs were used in 15 (12.3%) acutely ruptured aneurysms, of which 7 (46.7%) patients had a World Federation of Neurosurgical Societies (WFNS) grade of 4 or 5. There were 14 (11.5%) recurrent aneurysms previously treated with coiling (*n* = 9), intrasaccular flow disruption (*n* = 3), and stent-assisted coiling (*n* = 2). The aneurysm morphology was saccular in 100 cases (82.0%), fusiform in 12 (9.8%), blister-like in 6 (4.9%), and dissecting in 4 (3.3%). The mean aneurysm size was 8.3 ± 6.8 mm, with 12 (9.8%) being very large or giant (≥ 20 mm). The mean neck width was 4.7 ± 3.2 mm, and the mean dome to neck ratio was 1.4 ± 0.4. All but one aneurysm (99.1%) had a wide neck. Baseline aneurysm characteristics are summarized in Table 1.

**Table 1:** Baseline aneurysm characteristics. Pcom = posterior communicating artery, AchoA = anterior choroideal artery

Characteristic	Value (*n* = 122)
Unruptured, naïve aneurysms	93 (76.3%)
Recurrent aneurysms	14 (11.5%)
Acutely ruptured aneurysms	15 (12.3%)
*Aneurysm location*	
Internal carotid artery	
– Cervical segment	4 (3.3%)
– Paraophthalmic segment	67 (54.9%)
– Pcom segment	19 (15.6%)
– AchoA segment	4 (3.3%)
Anterior cerebral artery	2 (1.6%)
Basilar artery	11 (9.0%)
Vertebral artery	10 (8.2%)
Posterior cerebral artery	1 (0.8%)
Anterior inferior cerebellar artery	1 (0.8%)
Posterior inferior cerebellar artery	3 (0.8%)
*Aneurysm morphology*	
Saccular	100 (82.0%)
Fusiform	12 (9.8%)
Blister-like	6 (4.9%)
Dissecting	4 (3.3%)
Aneurysm size (mm)	8.3 ± 6.8 (range: 1.5–34)
Small (< 10 mm)	90 (73.8%)
Large (≥ 10 and < 20 mm)	20 (16.4%)
Very large (≥ 20 mm)	12 (9.8%)
Neck width (mm)	4.7 ± 3.2 (range: 1–20)
Dome-to-neck ratio	1.4 ± 0.4 (range: 1.0–3.2)
Parent artery diameter (proximal, mm)	4.1 ± 0.8 (range: 1.3–6.3)
Parent artery diameter (distal, mm)	4.0 ± 0.9 (range: 1.3–6.5)

### Treatment

**Fig. 1:**
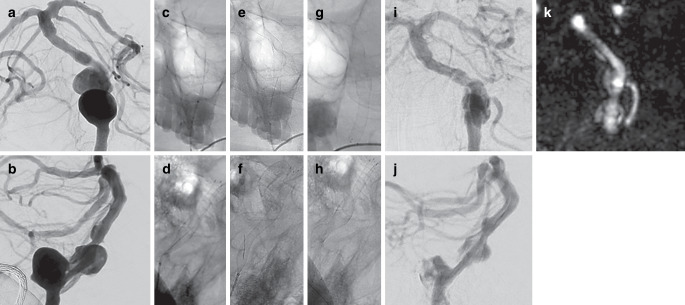
Digital subtraction angiography (DSA) shows a V4 aneurysm of the vertebral artery (18 mm), a sidewall aneurysm of the basilar artery (13 mm), and dysplastic basilar and posterior cerebral arteries in antero-posterior (**a**) and lateral (**b**) projections. The two aneurysms were treated with a 5.0 × 40 mm DED 2. Unsubtracted images (**c**–**h**) show the delivery of the DED and illustrate the fluoroscopic visibility of the entire device contour, even in the skull base region. During delivery, the DED did not unfold properly (**c** anteroposterior, **d** lateral), which was resolved by pushing the microcatheter (**c** anteroposterior, **d** lateral). After detachment, the DED showed good wall adaptation (**g** anteroposterior, **h** lateral). Eleven-month control DSA shows progressive aneurysm occlusion (**i** anteroposterior, **j** lateral; OKM B each). 37-month magnetic resonance angiography reconstructions show further improvement in aneurysm occlusion (k; OKM C, each)

**Fig. 2:**
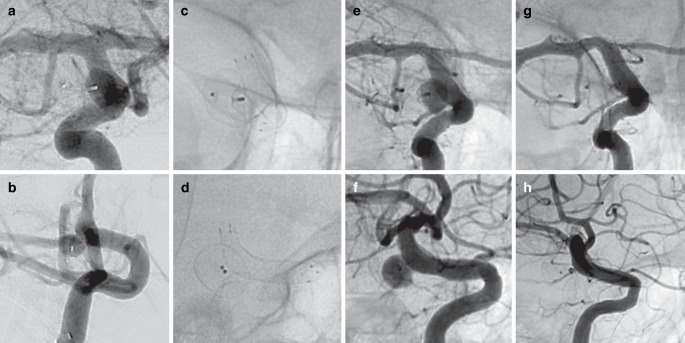
Digital subtraction angiography shows a recurrent paraophthalmic internal carotid artery aneurysm after previous implantation of a Woven Endobridge (WEB) in the anteroposterior (**a**) and lateral (**b**) views. The residual aneurysm was treated with a 4.5 × 15 mm DED 1, which was technically uneventful. Figures **c** + **d** show the contour of the DED and the WEB in unsubtracted images. The five-month angiographic control shows persistent perfusion of the aneurysm remnant (**e** + **f**), but the 18-month FU shows complete aneurysm occlusion (**g** + **h**)

Out of 103 procedures, DED 1 was used in 80 (77.7%) cases and DED 2 in 23 (22.3%) cases. The mean diameter of the DED was 4.7 ± 0.9 mm, with the 4.5 mm diameter being the most commonly used in 34 (33.0%) cases. However, all other available diameters were used, ranging from 2.5 to 8 mm, as shown in Table 2. The mean DED-to-parent artery ratio was 1.2 ± 0.1, indicating oversizing in all cases ranging from 4 to 90%. The 90% oversizing was performed in a dysplastic artery where the proximal and distal landing zones were of normal caliber. DED deployment was technically successful in all but one procedure. In this case, the DED failed to achieve sufficient wall apposition in a 15 mm paraophthalmic ICA aneurysm and was removed. The patient was subsequently treated with stent-assisted coiling. The mean number of FDs implanted per procedure was 1.1 ± 0.3, with more than one FD implanted in 7 (6.8%) procedures. A dysplastic Pcom aneurysm with a very broad base was treated with 3 FDs in telescoping technique, 2 DEDs and a FRED flow diverter (Microvention, Aliso Viejo, CA, USA). Additional coils were implanted in 5 (4.8%) aneurysms and secondary balloon angioplasty was performed in 6 (5.8%) cases. In one cervical fusiform aneurysm, an additional stent was implanted to compensate for proximal fish-mouthing of the implanted DED. A total of 107 side branches were covered, of which 106 (99.1%) remained patent at the end of the procedure. Details of the procedure are summarized in Table 2. Illustrative cases of DED implantation are shown in Figs. 1 and 2.

**Table 2:** Procedural characteristics

Characteristic	Value (*n* = 103)
Technical success	102 (99.0%)
*Number of DEDs*	
1	96 (93.2%)
2	6 (5.8%)
3	1 (1.0%)
*DED type*	
DED 1	80 (77.7%)
DED 2	23 (22.3%)
*DED diameter*	
Mean (mm)	4.7 ± 0.9
2.5 mm	3 (2.9%)
3.5 mm	8 (7.8%)
4.0 mm	16 (15.5%)
4.5 mm	34 (33.0%)
5.0 mm	20 (19.4%)
5.5 mm	14 (13.6%)
6.0 mm	3 (2.9%)
7.0 mm	3 (2.9%)
8.0 mm	2 (1.9%)
DED/parent artery diameter ratio	1.2 ± 0.1 (range: 1.04–1.9)
Adjunctive coiling	5 (4.8%)
Use of balloon	6 (5.8%)
Patent side branches at end of procedure	106/107 (99.1%)

Immediate contrast retention was observed in 95 (77.9%) aneurysms, of which 40 (32.8%) showed severe stasis (OKM 3) and 55 (45.1%) moderate stasis (OKM 2). In univariate analysis, treatment of recurrent aneurysms (HR: 9.0, 95%CI: 2.7–30.3, *p* < 0.001), larger aneurysm size (HR: 1.3, 95%CI: 1.1–1.6, *p* = 0.002), and larger parent vessel diameter (HR: 2.2, 95%CI: 1.2–4.3, *p* = 0.017) negatively correlated with immediate stasis. On multivariate analysis, recurrent aneurysms (HR: 5.7, 95%CI: 1.4–23.8, *p* = 0.018) and aneurysm size (HR: 1.4, 95%CI: 1.1–1.8, *p* = 0.009) were independently associated with lack of stasis in the aneurysm sac.

### Complications and Clinical Outcome

Procedural symptomatic complications occurred in 9 (8.7%) procedures, 8 (7.8%) of which were considered device-related. These were 2 (1.9%) major complications (1 major stroke, 1 intracranial hemorrhage with associated infarction) and 6 (5.8%) minor complications (6 minor strokes). The non-device related complication was transient cortical blindness in response to contrast. All strokes were considered thromboembolic and showed punctate infarcts on MRI. One patient had an mRS score of 2 at discharge, 3 had mRS 1, and 3 had mRS 0. The intracranial hemorrhage occurred during treatment of a very large (24 mm) paraophthalmic ICA aneurysm due to microwire perforation of the M2 segment, which was subsequently coil-embolized leading to infarction of the associated territory. The patient was transferred to a rehabilitation center with an mRS score of 4.

The procedural morbidity was 4.8% (5/104) and there was no mortality.

Besides the symptomatic complications, there were 5 technical asymptomatic events: There were 2 dissections, of which 1 cervical ICA dissection was treated with a stent and 1 VA dissection was not hemodynamically relevant. There was one apposition thrombus at the distal DED after deployment in an unruptured paraophthalmic ICA aneurysm. In another case, a dissecting paraophthalmic ICA aneurysm, control angiography after deployment showed thrombus formation within the FD. Both patients were on DAPT and had an adequate response. In both cases, the thrombus was dissolved with i.v. tirofiban. Finally, during the treatment of a paraophthalmic ICA aneurysm, there was a thromboembolic occlusion of the right A1 requiring stent implantation.

Of 25 patients who underwent post-interventional MRI up to 7 days after treatment, 11 (44.0%) showed ischemic lesions on diffusion-weighted imaging (DWI). Of these, 5 (20.0%) patients were symptomatic (see above).

There were no factors associated with procedural stroke in the univariate analysis. At clinical follow-up, there were no delayed complications.

### Angiographic Follow-up

Angiographic follow-up was available for 80 patients with 87 aneurysms. Angiographic results are detailed in Table 3.

**Table 3:** Angiographic results

	Immediate (end of procedure)	Short-term (1–6 months)	Mid-term (7–18 months)	Long-term (> 18 months)
Number of aneurysms	122	74	38	33
Follow-up period	–	4.9 ± 1.7	12.4 ± 4.5	34.9 ± 14.3
Total filling (> 95%) (OKM A)	113 (92.6%)	16 (21.6%)	1 (2.6%)	1 (3.0%)
Subtotal filling (5–95%) (OKM B)	7 (5.7%)	12 (16.2%)	4 (10.5%)	0 (0%)
Entry remnant (< 5%) (OKM C)	0 (0%)	6 (8.1%)	6 (15.8%)	7 (21.2%)
Complete occlusion (OKM D)	2 (1.6%)	40 (54.1%)	27 (71.1%)	25 (75.8%)

*OKM* O’Kelly-Marotta scale

Complete and favorable occlusion rates were 54.1 and 62.2% with short-term FU (mean: 4.9 ± 1.7 months), 71.1 and 86.9% with mid-term FU (mean: 12.4 ± 4.5 months), and 75.8 and 97.0% with long-term FU (mean: 34.9 ± 14.3 months), respectively. The only aneurysm with inadequate occlusion (OKM A or B) at long-term FU was a 7.3 mm paraophthalmic ICA aneurysm with the ophthalmic artery originating directly from the aneurysm sac of a 30-year-old man treated with 2 overlapping DEDs.

Twenty-five (54.1%) patients with complete occlusion (OKM D) at initial follow-up had additional mid- or long-term follow-ups, and in all cases the aneurysm remained completely occluded.

Of 34 patients with incomplete occlusion at short-term follow-up, 17 (50.0%) patients had additional follow-up, with occlusion status improving in 14 (82.4%) and remaining stable in 3 (17.6%). Of 11 patients with incomplete occlusion at mid-term follow-up, 7 (63.6%) had long-term follow-up, with occlusion status improving in 4 (36.4%) and remaining stable in 3 (27.3%). Table 4 compares baseline characteristics and procedural specifics of aneurysms with persisting incomplete occlusion and with progressive occlusion between mid- and long-term follow-up. Incomplete occlusion at long-term follow-up tended to be associated with large aneurysm neck width and large DED diameter/parent artery diameter.

**Table 4:** Baseline and procedural characteristics of aneurysms with persisting incomplete occlusion and with progressive occlusion between mid- and long-term follow-up

Characteristic	Persisting incomplete occlusion (*n* = 3)	Progressive occlusion (*n* = 4)	P‑value
Unruptured, naïve aneurysms	3 (100%)	3 (75%)	
Recurrent aneurysms	0 (0%)	1 (25%)	
Acutely ruptured aneurysms	0 (0%)	0 (0%)	
*Aneurysm location*			
Internal carotid artery	–		
– Paraophthalmic segment	1 (33%)	1 (25%)	
– Pcom segment	0 (0%)	1 (25%)	
– Basilar artery	2 (67%)	1 (25%)	
– Vertebral artery	0 (0%)	1 (25%)	
Saccular morphology	3 (100%)	4 (100%)	
Aneurysm size (mm)	16.1 ± 8.9	10.3 ± 4.7	
Neck width (mm)	12.4 ± 5.3	6.6 ± 3.4	0.04
Dome-to-neck ratio	1.1 ± 0.3	1.5 ± 0.3	
Parent artery diameter (proximal, mm)	5.0 ± 0.1	4.2 ± 0.3	0.02
Parent artery diameter (distal, mm)	4.9 ± 0.2	4.2 ± 0.4	0.04
Multiple DEDs	0 (0%)	0 (0%)	
DED 2	0 (0%)	0 (0%)	
Mean DED diameter (mm)	5.5 ± 0	4.8 ± 2.9	0.02
DED/parent artery diameter ratio	1.1 ± 0.03	1.2 ± 0.1	
Adjunctive coiling	1 (33%)	0 (0%)	
Use of balloon	0 (0%)	0 (0%)	
Immediate stasis	3 (100%)	3 (75%)	

Only significant *p*-values are shown

Overall, in patients with multiple follow-ups, aneurysm occlusion improved in 8/26 (30.1%) aneurysms between short- and mid-term FU, in 13/28 (46.4%) cases between short and long-term FU, and in 4/16 (25.0%) aneurysms between mid-term and long-term FU. The remaining cases showed stable occlusion with no recanalization.

**Fig. 3:**
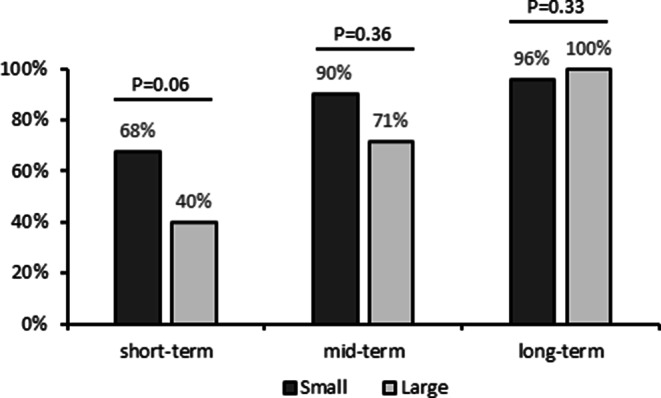
Favourable occlusion rates of small (< 10 mm) and large (≥ 10 mm) aneurysms treated with DED. (Short-term FU was 1–6 months, medium-term was 7–18 months, and long-term was > 18 months after treatment)

Aneurysm occlusion rates of large aneurysms were lower than those of small aneurysms at short-term follow-up, but improved over time and were comparable at long-term follow-up, as shown in Fig. 3. Larger neck width (HR: 1.2, 95%CI: 1.0–1.5, *p* = 0.029) and larger parent vessel diameter (HR: 4.4, 95%CI: 1.6–11.8, *p* = 0.003) correlated with unfavorable short-term occlusion. Parent artery diameter remained independently associated with unfavorable occlusion in multivariable analysis (HR: 1.1, 95%CI: 1.0–1.3, *p* = 0.032).

Seven (9.5%) in-stent stenoses were observed at short-term FU, of which 2 were considered moderate and 5 were considered mild. One moderate stenosis progressed to a severe stenosis at follow-up and remained stable. Interventional retreatment was not required after interdisciplinary case discussion. Four stenoses remained stable at follow-up and 2 mild stenoses were lost to follow-up. Medical management of the moderate and severe stenoses consisted of continuation and dose escalation of dual anti-platelet therapy (ASA 100 mg and clopidogrel 150 mg). Cases with mild in-stent stenoses received ASA as usual.

## Discussion

The present study demonstrates progressive occlusion of aneurysms treated with DEDs during long-term follow-up, with favorable occlusion (OKM C + D) achieved in 62% at a mean of 5 months, 87% at 12 months, and 97% at 35 months. Aneurysm treatment was associated with a feasibility rate of 99%. Complication rates were low with a 2% rate of major events and 5% rate of procedural morbidity, and there was no mortality.

### Aneurysm Occlusion

The DED has been evaluated in numerous single-center series and several multicenter studies, but a systematic analysis of the long-term safety and efficacy of this device is not yet available. Among the multicenter studies, complete occlusion rates were 81% at 6 months and 89% at 12 months in the study by Trivelato et al., 75% at 6 months and 83% at 12 months in the study by Pinana et al., and Taschner et al. reported favorable occlusion in 89% at 12 months [14, 16, 19]. Piano et al. reported complete occlusion in 62% at 6 months and 75% at 18 months [20]. The angiographic results of the present study corroborate these data and additionally show that progressive occlusion occurs beyond 12 months after DED implantation, reaching a favorable occlusion rate of 97% at a mean follow-up of 35 months. Specifically, aneurysm occlusion improved in 46% between short and long-term follow-up and in 25% between mid- and long-term follow-up, with no recanalization. Aneurysm occlusion of large aneurysms was inferior to that of small aneurysms at short-term follow-up, confirming aneurysm size as a risk factor for incomplete aneurysm occlusion after flow diversion [21]. However, the analysis showed comparable results at mid- and long-term follow-up, confirming the efficacy of DED implantation for large aneurysms.

The only case of unfavorable occlusion at long-term follow-up was a paraophthalmic ICA aneurysm in which the ophthalmic artery originated directly from the sac. A vessel arising from the sac may maintain flow due to the pressure gradient across the ostium and may prevent progressive aneurysm thrombosis, reflecting a risk factor for treatment failure [22].

The angiographic results of the DED are consistent with the available studies of the first-generation PED, which is the best-studied FD in the literature. In the PEDESTRIAN registry, Lylyk et al. reported complete occlusion rates of 76% at 1 year, 93% at 3 years, and 96% beyond 5 years [23]. In the PUFS study, complete occlusion rates were 87% at 1 year, 93% at 3 years, and 95% at 5 years [24]. These results indicate that the surface modification of the DED does not appear to adversely affect neointimal growth along the aneurysm neck and subsequent progressive thrombosis of the aneurysm sac.

There is an ongoing debate as to whether additional coiling or multiple stent implantation improves the efficacy of FD treatment. In the meta-analysis by Cagnazzo et al., additional coiling was not associated with improved occlusion rates, but was associated with an increased risk of procedural complications [25]. In contrast, Wang et al. reported improved occlusion with additional coiling for aneurysms > 7 mm with similar complication rates, whereas complication rates were increased for aneurysms ≤ 7 mm [26]. Similarly, the implantation of multiple FDs did not significantly increase the occlusion rates in the meta-analysis by Cagnazzo et al. and the Chalouhi et al. study, but increased the complication rate in both studies [25, 27]. Nevertheless, in selected cases, such as ruptured, large, or blister aneurysms, additional coiling or multiple stent deployment may be performed to assist intrasaccular thrombus formation or to provide immediate occlusion to prevent (re)rupture. In the present study, a wide aneurysm neck and a large parent vessel diameter correlated with long-term incomplete occlusion, justifying the need for additional coiling of these aneurysms. The fate of these aneurysms could be studied in a very long-term analysis.

The authors prefer to treat aneurysms with a single FD without additional coiling because this procedure is time and cost efficient and reduces procedural complications. Even in ruptured aneurysms, there is no clear evidence that a single FD carries a higher risk of rupture than multiple FDs or FD + coiling [28]. Moreover, the improved porosity of advanced FDs may contribute to immediate stasis and improved occlusion.

In addition to inadequate intra-aneurysmal thrombosis, excessive neointimal growth is another concern associated with intracranial stent or FD treatment. Overall, the incidence of in-stent stenosis is underreported in the literature. Among the articles focusing on in-stent stenosis, in-stent stenosis rates were reported in 54% for the PED by You et al. and in 44% for the SILK (Balt Extrusion, Montmorency, France) by Essbaiheen et al. [18, 29] Although in-stent stenoses are mild in the majority of cases and usually improve over time, neointimal hyperplasia is associated with endothelial cell and platelet activation, which can be a source of thromboembolism or lead to parent vessel occlusion, potentially causing delayed ischemic stroke [30]. To avoid this risk, we continue DAPT in cases with in-stent stenosis, as suggested by other authors, while reserving balloon angioplasty or stenting for severe or symptomatic cases [31]. In the present study, in-stent stenosis was observed in 9.5%, which is lower than the rates reported for PED and SILK. None of these stenoses were symptomatic. Although these data suggest that surface modification of the DED may reduce the risk of in-stent stenosis, a definitive conclusion cannot be drawn without a systematic, comparative, core-laboratory-adjusted analysis.

### Safety

Procedural thromboembolic complications occur due to the high metal coverage of FD and represent a real concern for flow diversion. For PED, major ischemic stroke and morbidity rates were 4.7 and 8.4% in the IntrePED study, 4.7 and 6.8% in the ASPIRe study, and 6 and 9% in the meta-analysis by Brinjikji et al.[5, 6, 32] Advanced FDs have undergone structural and delivery system modifications to reduce the thrombogenic potential of the treatment. These modifications are primarily aimed at reducing the metal content, for example by introducing low-profile devices and delivery systems. In the present study, major ischemic stroke occurred in 2%, which is lower than reported in most studies of the PED. Similarly, major complications for Derivo were 5% in the Trivelato study, 4% in the Pinana study, 2% in the Lourenco study, and 3% in the Taschner study [14, 16, 33, 34].

In addition to ischemic stroke, FD is associated with a higher risk of silent ischemic lesions that require endovascular coiling. Overall, ischemic lesions were found in 44% of patients on post-interventional MRI, which is within the lower range of the 95% CI (46–85%) reported for unselected FD types in the meta-analysis by Bond et al. and within the 95% CI for coiling (33–56%) [35]. However, it should be noted that post-interventional MRI was performed only in selected cases (symptomatic and complex cases), which may result in even lower rates of ischemic lesions in unselected patients, which needs to be evaluated in a prospective study.

Besides the occurrence of acute ischemic stroke, there is an additional risk of delayed thromboembolic complications, particularly after switching from dual antiplatelet therapy to monotherapy. In this context, Hwang et al. reported a delayed ischemic stroke rate of 3.5% within two months after switching to monotherapy after stent assisted coiling [36]. In this long-term study, we did not observe any delayed adverse events, particularly after switching from dual antiplatelet therapy to aspirin monotherapy.

### Limitations

The major limitations of the study are its single-center and retrospective design. There is a notable overrepresentation of patients with ICA aneurysms, including few cervical ICA aneurysms. Clinical and angiographic follow-up is lacking in several cases, which may bias the results, especially for incompletely occluded aneurysms where further follow-up is not available. In addition, the majority of included aneurysms were small, which has implications for occlusion rates, as larger aneurysms are at risk for incomplete occlusion and retreatment [21]. Despite promising mid-term safety and aneurysm occlusion rates, further studies are required to define the long-term efficacy of the DED.

## Conclusions

This study demonstrates progressive aneurysm occlusion after DED treatment beyond a 12-month FU period, with complete and favorable occlusion achieved in 76 and 97% at 35 months. The data also highlight the safety of DED treatment with a major ischemic stroke rate of 1% and the absence of delayed thromboembolic complications. These findings underscore the potential of FDD surface modification to improve procedural safety while maintaining good angiographic outcomes. The results warrant further clinical use of the DED and further investigation of this device and other advanced FDs, particularly regarding the role of antithrombotic surface modification.
